# Regulation of Magnetocaloric Effect in Ni_40_Co_10_Mn_40_Sn_10_ Alloys by Using a Homemade Uniaxial Strain Pressure Cell

**DOI:** 10.3390/ma15124331

**Published:** 2022-06-18

**Authors:** Kaiming Qiao, Yuhang Liang, Shulan Zuo, Cheng Zhang, Ziyuan Yu, Yi Long, Fengxia Hu, Baogen Shen, Hu Zhang

**Affiliations:** 1 School of Materials Science and Engineering, University of Science and Technology Beijing, Beijing 100083, China; kmqiao@ustb.edu.cn (K.Q.); g20208410@xs.ustb.edu.cn (Y.L.); yuziyuan95@163.com (Z.Y.); longyi@ustb.edu.cn (Y.L.); 2 School of Materials Science and Engineering, Beihang University, Beijing 100191, China; zuoshulan@buaa.edu.cn; 3 Beijing National Laboratory for Condensed Matter Physics, Institute of Physics, Chinese Academy of Sciences, Beijing 100190, China; zhangcheng@iphy.ac.cn (C.Z.); fxhu@iphy.ac.cn (F.H.); shenbg@iphy.ac.cn (B.S.)

**Keywords:** magnetocaloric effect, uniaxial strain, Heusler alloys

## Abstract

In this study, a homemade uniaxial strain pressure cell was designed to be directly used in the standard magnetometers whereby the magnetic properties of samples subjected to a uniaxial strain and magnetic field were characterized. Its feasibility has been demonstrated by the uniaxial strain control of the phase transition and magnetocaloric effect in Ni_40_Co_10_Mn_40_Sn_10_ (NCMS) alloys. With the assistance of a uniaxial strain of ~0.5%, the cooling temperature span of NCMS alloys is broadened by 2 K, and the refrigeration capacity under a 3 T magnetic field change increases from 246 to 277 J/kg. This research provides not only direct experimental assistance for the tuning of phase transition by the uniaxial strain but also possibilities for studying the coupled caloric effect in first-order phase transition materials under a combined uniaxial strain and magnetic field by the thermodynamic analysis.

## 1. Introduction

Solid-state refrigeration has received extensive attention as a substitute technology for refrigeration through gas compression owing to its environmentally harmless and energy-saving advantages [1,2,3,4,5,6,7,8,9,10,11]. Seeking a suitable refrigerant is one of the most important problems faced in the application of solid-state refrigeration technology [12]. From the discovery of Gd_5_(Si,Ge)_4_ [1], the magnetocaloric effect (MCE) of first-order phase transition materials has gained a lot of attention as a prospective candidate material for magnetic refrigeration. As the research further develops, various problems such as the narrow cooling temperature span [13,14,15] and large hysteresis loss [16,17] in these materials gradually emerge, limiting their application. Great efforts have been made to solve these problems by doping [18,19] or introducing multi-field stimuli [20,21,22]. Interestingly, when multi-fields are introduced, not only can the cooling temperature span be broadened, but also a multi-caloric response will occur due to the interplay between structural and magnetic degrees of freedom [23]. In recent years, multi-field stimuli have been widely used to control the phase transition and caloric effect, especially elastocaloric effect (eCE) and MCE, in materials with first-order phase transition [24,25].

Ni-Co-Mn-Sn Heusler alloys are a typical class of first-order phase transition materials simultaneously exhibiting both ferromagnetic order and ferroelastic order. Thus, both stress and magnetic field are able to induce the phase transition between ferromagnetic austenite (A) at high temperature and weak magnetic martensite (M) at low temperature while the transition direction under magnetic field and stress field is opposite. Consequently, both MCE and eCE exist in these materials and the sign of these two caloric responses is reverse. By introducing a combined magnetic field and uniaxial stress, a multi-caloric effect (MCE and eCE) covering a broad temperature span can be obtained in these alloys [24,26]. It is noted that in previous studies the caloric effect under uniaxial pressure and the magnetic field is usually characterized by direct measurement of adiabatic temperature change or quasi-direct method of calorimetric measurements [24,25,26,27]. Nevertheless, the ideal adiabatic condition is technically difficult to be fulfilled especially for the sample with a small size, and it puts forward higher requirements for magnets to couple with uniaxial stress. It is more accurate and easier to characterize MCE with standard magnetometers and both caloric responses are strongly dependent on the temperature-dependent magnetization curves of the materials. Thus, it is necessary to find a way to characterize the caloric effect subjected to the coupled magnetic field and uniaxial stress by standard magnetometers. Moreover, the direct measurement of magnetization under coupled magnetic field and strain makes it possible to construct phase diagrams of magnetic, strain (stress), and temperature evolution for first-order phase transition materials. From the above phase diagrams, the coupled caloric effect can be obtained by the thermodynamic analysis [23].

In this context, we propose to design and fabricate a uniaxial strain pressure cell, which can be directly used in the commercial Versa-lab magnetometers. Thus, magnetic properties in materials under the combined magnetic field and uniaxial stress can be directly measured. This makes it possible to construct phase diagrams of magnetic, pressure, and temperature evolution for first-order phase transition materials, and then the multi-caloric effect (especially coupled caloric effect) can be obtained by the thermodynamic analysis. Moreover, by utilizing this pressure cell, the control of phase transition and MCE in Ni_40_Co_10_Mn_40_Sn_10_ (NCMS) alloys was studied. The NCMS alloys’ refrigeration temperature span can be effectively widened by the in situ uniaxial strain, which is highly important for the use of materials with first-order phase transition.

## 2. Methods

**Figure 1:**
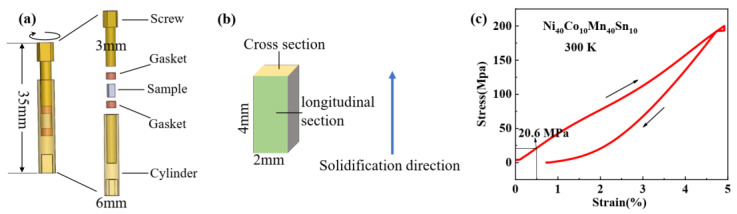
(**a**) The overall and exploded schematic diagram of a home-built uniaxial strain pressure cell, where the dimensions and descriptions of each part are marked. (**b**) Schematic of the NCMS sample, where its size and direction are marked. (**c**) Compressive stress-strain curves (red line) measured with a low strain rate of 1.3 × 10^−4^ s^−1^ at 300 K and the arrows indicate the loading and unloading process.

Nominal compositional polycrystalline Ni_40_Co_10_Mn_40_Sn_10_ alloys were synthesized by arc melting in the argon atmosphere, where pure Ni, Co, Mn, and Sn metals (more than 99.9%) were used. To compensate for the weight loss of Mn, additional Mn (5 wt%) was added during arc melting. The obtained ingots were sealed in a quartz tube with high-purity argon environment, then annealed for 96 h at 1123 K before being quenched into ice water. The ingots were cut into pieces with a size of 2 × 2 × 4 mm^3^. X-ray diffraction (XRD) utilizing Cu Kα radiation was measured to evaluate the crystal structure evolution of the NCMS alloy from 300 K to 100 K during the cooling process by a Rigaku Smart Lab diffractometer. The microstructure and elemental analysis measurements were carried out by field-emission scanning electron microscope (SEM, ZEISS EVO 18, Oberkochen, Germany) coupled with X-ray energy-dispersive spectroscopy (EDS). For this measurement, the specimens were mechanically polished and chemically etched in a solution of 20 mL HCl + 5 g FeCl_3_ − 6H_2_O + 96 mL ethanol for approximately 45 s. The magnetic characteristics of the sample were determined using Quantum Design Inc. (San Diego, CA, USA) cryogen-free cryocooler-based physical property measurement equipment (model Versa-Lab). The uniaxial strain was applied in situ by the homemade pressure cell as shown in Figure 1.

## 3. Results and Discussion

Figure 1a illustrates the schematic diagram of a homemade uniaxial pressure cell, which can be directly used in the commercial physical property measurement system. To avoid the influence of the magnetic signal in the pressure cell, brass was used to produce the pressure cell. The shape of the pressure cell is designed to match the cylinder and the size of the outer diameter for the pressure cell is limited to 6 mm, as schematically shown in Figure 1a, owing to the size limitation of the VSM coil set (i.e., 6.3 mm) in our Versa-lab. To make the size of the sample as close as possible to that indirect measurement of eCE, the wall thickness of the pressure cell is minimized to 1.5 mm and the inner diameter is 3 mm with internal thread. The uniaxial strain can be applied to the sample by turning the screw, as pointed out by the curved arrow in Figure 1a. To reduce the torsional strain of the sample with the rotation of the screw, two smooth brass cylindrical gaskets with a diameter of 2.5 mm are placed above and below the sample. Different from the direct measurement of adiabatic temperature change at the universal testing machine with the stress being a constant, the strain is a constant in this pressure cell during the magnetic measurements. To test the feasibility of the homemade uniaxial pressure cell, NCMS alloys prepared by arc melting were used. It is worth noting that the texture structure may be generated with columnar grains along the direction of solidification due to the large temperature gradient from the top to the bottom of the ingot in the arc melting process and such grain distribution microstructure highly affects mechanical properties of the materials [28,29]. As shown in Figure 1b, the NCMS sample with a size of 2 × 2 × 4 mm^3^ was cut from the button ingot along the solidification direction. Before the magnetic measurements, we first carefully studied the crystal structure and microstructure of NCMS alloys. To quantitatively determine the applied strain and stress, the cell length will be measured by the spiral micrometer under conditions without sample, with samples under no strain, and with samples under uniaxial strain. Then, the uniaxial strain is determined to be about 0.5% in the current study. The corresponding stress can be estimated from the stress-strain curves at room temperature as shown in Figure 1c. It can be seen from the stress-strain curves that in the loading process, the stress is about ~20 MPa under a uniaxial strain of 0.5%. It should be noted that the samples used for the measurement of the stress-strain curve and magnetic properties have the same size, which can eliminate the size effect.

**Figure 2:**
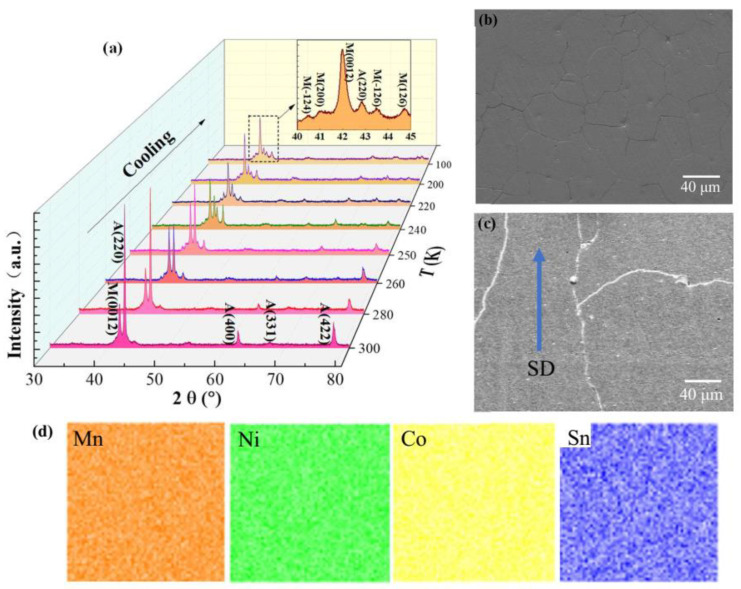
(**a**) In situ temperature-variable XRD patterns of NCMS alloy during the cooling process and partial XRD peaks of the 6M martensite at 100 K are enlarged in the inset. Secondary electron scanning electron microscopy (SEM) images of NCMS ingot in the (**b**) cross-section and (**c**) longitudinal section, where the solidification direction is indicated by the blue arrow. (**d**) EDS mapping for NCMS alloy at room temperature.

Figure 2a shows temperature-dependent XRD patterns of NCMS alloy during the cooling process. From the XRD pattern at 300 K, one can see that the B2 cubic austenite phase is dominant with a lattice parameter of *a* = 5.984 Å, while a small amount of martensite phase coexists as indicated by the peak on the left side of the strongest austenite peak. As the temperature decreases to 280 K, the intensity of the peaks associated with the austenite phase decreases. The intensity of martensite peaks becomes stronger as the temperature drops, while the (220)_A_ peak of the austenite phase steadily diminishes. However, a minor quantity of austenite phase remains at 100 K, as seen in the magnified picture of the XRD pattern at this temperature. According to the careful indexations in the inset of Figure 2a, a six-layered modulated (6M) monoclinic structure can be confirmed in the martensite. The crystal structure evolution shows that the manufactured NCMS alloy has a typical martensitic transition.

Figure 2b,c show the secondary electron scanning electron microscopy (SEM) image of NCMS alloys in the cross and longitudinal sections as marked in Figure 1b, respectively. It is clear to observe that the grain sizes and shapes are very different for the SEM images along with different directions. The equiaxed crystal with a grain size of tens of microns occurs in the cross-section while long strip grain appears in the longitudinal section with sizes of hundreds of microns along the strip direction. This kind of long strip grain induced by the temperature gradient in solidification will highly affect the mechanical properties of NCMS alloys [28,29]. To study the elemental dispersion for NCMS alloy, the composition maps are performed by EDS and the EDS mapping images of Mn, Ni, Co, and Sn elements are shown in Figure 2d. The EDS analysis does not show measurable chemical inhomogeneity, which confirms the homogeneity of composition in NCMS alloys.

**Figure 3:**
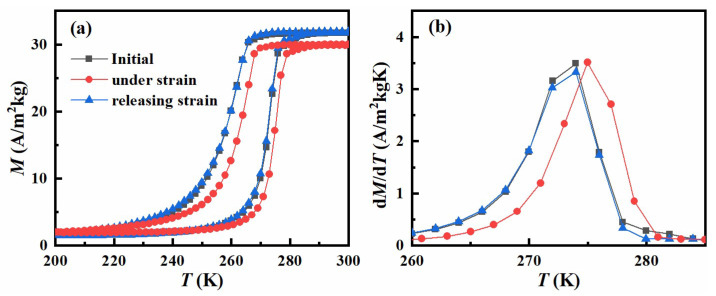
(**a**) Temperature-dependent magnetization curves in the cooling and heating process and (**b**) the corresponding d*M*/d*T-T* curves in the heating process under a magnetic field of 0.01 T for NCMS alloys in the initial state, compressive strain state, and after releasing the strain, respectively.

To verify the martensitic transition in Ni_40_Co_10_Mn_40_Sn_10_ alloys and its modulation by our homemade uniaxial pressure cell, the thermomagnetic curves under a magnetic field of 0.01 T in the initial and uniaxial compressive strain state are measured and the corresponding results are exhibited in Figure 3. Here, the strain determined by the spiral micrometer is about 0.5%, and thus, the corresponding stress is estimated by the stress-strain curves at room temperature to be about ~20 MPa, as shown in Figure 1c. Both the *M-T* curves in the compressive state and the initial state show typical characteristics of the magnetic transition from weak magnetic martensite to ferromagnetic austenite in the heating process, indicated by a sudden jump of magnetization across the transition from 265 K to 280 K. The transition temperature is located at 272 K in the heating process in the initial state and a thermal hysteresis about 14 K occurs for NCMS alloys, which can be seen in the d*M*/d*T-T* curves as shown in Figure 3b. When a uniaxial compressive strain was applied to the NCMS alloys, the magnetization decreases and the *M-T* curve moves to a higher temperature. This confirms the effectiveness of our homemade uniaxial strain cell in tuning the magnetic properties in situ. The reduction of magnetization and shift of transition temperature can be ascribed to the volume and the atom’s distance change under the compressive strain [30]. The Mn-Mn distance has a significant impact on the magnetic characteristics of Heusler alloys. Under compressive strain, the Mn-Mn distance in NCMS alloys is shortened, resulting in an increase in antiferromagnetic interactions. As a result, under compressive strain, the magnetization drops. The compressive strain, like hydrostatic pressure, reduces the volume and brings the atoms closer together, promoting bonding and orbital hybridization in NCMS alloys [30]. As a result, more thermal energy is required to cause the austenitic transition, resulting in a shift in temperature under compressive strain. When a uniaxial strain is applied, the transition temperature shifts about 2 K during the heating process and about 4 K during the cooling process. Such a difference is similar to the phenomenon under the electric-field-induced strain state in the ferromagnetic/ferroelectric composite [13], which may be caused by the combined effect of temperature and strain. In the heating process, the compressive strain stabilizes the martensite and the transition temperature shifts towards a higher temperature. On the contrary, the increase in temperature promotes the austenite, and so this opposite effect reduces the increase of transition temperature. On the other hand, during the cooling process, both compressive strain and the decrease of temperature favor the martensite, leading to the larger shift of transition temperature. After releasing the compressive strain, the *M-T* curve is restored to the initial state, as shown in Figure 3. Moreover, the thermal hysteresis can be decreased from 14 to 10 K by utilizing a compressive strain in the cooling process while removing the compressive strain in the heating process. This fact suggests that if a larger compressive strain is applied, the thermal hysteresis could be reduced by controlling the compressive strain during the heating/cooling process. Although two smooth polished cylinders were used in the cell to reduce the friction between the sample and the screw, a torque may still occur, leading to a crack of the sample with a larger strain applied. In addition, from the SEM image, we can see that long strip-like grains appear in NCMS alloys along the direction of applying uniaxial strain. When a torque is exerted on the sample, it is easy to crush along the grain boundary. If the sample and uniaxial strain cell are improved, a larger strain may be applied so that the transition temperature could shift to a higher temperature. Thus, a completely reversible phase transition could be achieved.

**Figure 4:**
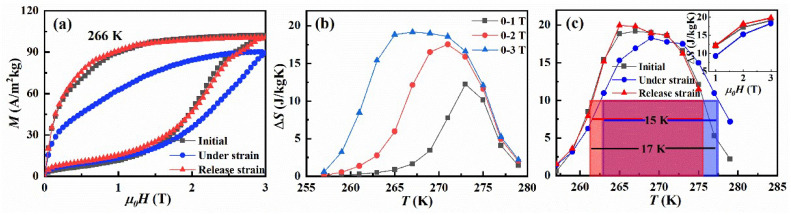
(**a**) Isothermal magnetization curves at 266 K. (**b**) Temperature-dependent entropy change under different magnetic fields for NCMS alloys in the initial state. (**c**) Comparison of the Δ*S*-*T* curves under a magnetic field change of 0–3 T for the cases in the initial state, compressive strain state, and releasing strain state, where the cooling temperature span is marked by the shadow area. The inset shows the comparison of the magnetic-field-dependent entropy change for the above three cases.

The isothermal magnetization curves spanning the transition were obtained in the loop mode to explore the effect of in situ uniaxial strain on the magnetocaloric effect of NCMS alloys [31]. As a representative, the *M-μ_0_H* curves in the initial and uniaxial compressive strain state at 266 K are exhibited in Figure 4a. The magnetic-field-induced metamagnetic transition can be seen from the *M-μ_0_H* curves in the initial state. The shape of *M-μ_0_H* curves remains unaltered when a uniaxial compressive strain is applied. However, the magnetization reduces significantly and the critical magnetic field of the metamagnetic transition increases under the compressive strain. The reduction of magnetization is caused by the reduction of Mn-Mn distance, whereby the variation of the exchange interaction in NCMS alloys is induced by the compressive strain. Meanwhile, as previously indicated, compressive strain stabilizes weak magnetic martensite, necessitating a higher critical magnetic field to induce the metamagnetic martensite-austenite transition. The obtained *M*-*μ_0_H* curves were used to calculate the magnetic entropy change by the Maxwell relationship [32] and the associated Δ*S*-*T* curves are displayed in Figure 4b. The accuracy of the entropy changes determined from *M*-*μ_0_H* curves is about 20%. It can be seen that as the magnetic field increases, the peak of Δ*S*-*T* curves broadens to the lower temperatures and the peak value gradually increases, evolving into a platform. This behavior gives rise to the magnetic-field-induced inverse martensitic transformation. With the magnetic field increasing, the amount of martensitic phase transforming into austenitic phase gradually increases so that the entropy changes. When the entropy change caused by the magnetic-field-driven phase transition saturates, the entropy change stops rising, resulting in a platform in the Δ*S*-*T* curves at high magnetic fields. With a magnetic field change of 0–3 T, the peak values of Δ*S*-*T* curves in the initial condition reached 19 J/kg K. While the peak value of Δ*S-T* curve reduces to 18 J/kg K under the uniaxial strain condition at the same magnetic field change, and the peak moves 2 K higher as shown in Figure 4c, which can be attributed to fluctuation of the exchange interaction generated by the compressive strain. The full width at half maximum (FWHM) of Δ*S*-*T* curves in both the initial and uniaxial strain state reaches 15 K, while the cooling temperature span in the initial state (261–276 K) is different from that in the uniaxial strain state (263–278 K) as indicated by the shadow areas in Figure 4c. It can be seen from the above |Δ*S*(*T*)| curves, the refrigeration temperature span is broadened to 17 K by applying uniaxial strain above 273 K and removing the strain below 273 K. Accordingly, the evaluated refrigeration capacity (RC) under a magnetic field change of 3 T can be enhanced from 246 to 277 J/kg by coupling the uniaxial strain with the magnetic field. Consequently, the phase transition and MCE in NCMS alloys can be tuned and directly measured in the standard magnetometers with the magnetic field and uniaxial strain applied simultaneously.

**Figure 5:**
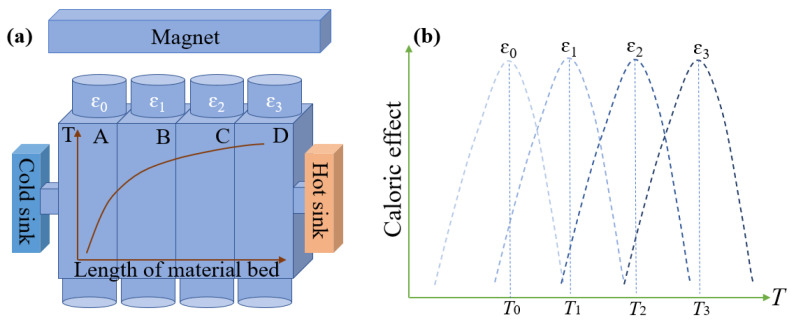
(**a**) The schematic diagram of the temperature gradient in the material bed, where the temperature gradient is displayed, where T indicate the temperature of the refrigerates and A, B, C, D indicate refrigerants at four different positions in the material bed. (**b**) The optimal working temperature of refrigerants tuned by the uniaxial strain.

As the one most used in the present magnetic refrigeration prototype, the active magnetic refrigerator (AMR) cycle is a promising refrigeration cycle with high energy efficiency [33,34], while a temperature gradient will be generated in the material bed from the hot to cold ends. Figure 5a exhibits the schematic diagram of AMR, including a hot sink, a cold sink, and a refrigerant’s bed (e.g., NCMS). There are usually four stages in a complete AMR cycle, namely, magnetization, hot blow, demagnetization, and cold blow. In the hot/cold blow process, a temperature gradient in the materials bed between the hot sink and the cold sink is established as shown in Figure 5a. Refrigerants at different positions work at different temperatures (*T*_0_ for refrigerate A, *T*_1_ for refrigerate B, *T*_2_ for refrigerate C, *T*_3_ for refrigerate D) so that not all of them can work in the optimal temperature, which results in a decrease of the cooling performance. Although refrigerates with different phase transition temperatures can be used, the cost of material preparation will have a substantial increase. In addition, the working temperature of refrigerates will change with the temperature change of the hot and cold end. It is difficult to control the working temperature of every position. If a uniaxial strain could assist in the AMR cycle, only one material is needed and the phase transition temperature of this material can be continuously adjusted to the optimal temperature by tuning the uniaxial strain. Hence, the cooling performance can be enhanced and the cost of material preparation can be saved. For example, *T*_0_ is the optimal temperature for NCMS alloys in the initial state and the temperature of the refrigerants in the material bed varies from *T*_0_ to *T*_3_. If suitable uniaxial strains (ε_0_, ε_1_, ε_2_, and ε_3_) are applied to different refrigerants (A, B, C, D), the temperature of each refrigerant could be optimized to be in line with the increasing temperatures in the material bed as shown in Figure 5b. As a result, the cooling performance of the AMR cycle could be improved with the assistance of uniaxial strains.

## 4. Conclusions

In conclusion, we have manufactured a uniaxial pressure cell, which can be directly used in the standard magnetometers. Thus, the magnetic properties under the combination of uniaxial strain and magnetic field can be directly characterized by the standard magnetometers. This is highly important for the research on the interplay between structural and magnetic degrees of freedom and the coupling effect in the first-order phase transition materials. The feasibility of this cell was demonstrated in the regulation of the phase transition and magnetocaloric effect in NCMS alloy. The cooling temperature span of Ni_40_Co_10_Mn_40_Sn_10_ alloys can be broadened by 2 K and the evaluated RC values under a magnetic field change of 3 T are enhanced from 246 to 277 J/kg with the assistance of a uniaxial strain of about 0.5%. This research provides not only direct experimental assistance for the tuning of phase transition by the uniaxial strain but also possibilities for research on the coupling effect under the combination of uniaxial strain and magnetic field in the first-order phase transition materials.

## Data Availability

The data presented in this study are available on request from the corresponding author. The data are not publicly available due to restrictions privacy.
